# FXTAS and the Spectrum of *FMR1* Premutation‐Associated Phenotypes in Latin America: A Scoping Review

**DOI:** 10.1002/mdc3.70617

**Published:** 2026-04-05

**Authors:** Amy Schmidmajer, Salomón Páez‐García, Santiago Martínez, Jacobo Ramírez‐Triana, Kevin O'Hara‐Veintimilla, Andrés Ricaurte‐Fajardo, Catalina Cerquera‐Cleves

**Affiliations:** ^1^ Semillero de Neurociencias y Envejecimiento, Ageing Institute, Medical School Pontificia Universidad Javeriana Bogotá Colombia; ^2^ Unidad de Neurología, Departamento de Neurociencias Hospital Universitario San Ignacio Bogotá Colombia; ^3^ Psychogeriatric Unit, Clínica Josefina Arregui Alsasua Spain; ^4^ Division of Molecular Imaging and Therapeutics, Department of Radiology, Weill Cornell Medicine New York City NY USA; ^5^ Neuroscience Research Program CHU de Québec‐Université Laval Research Center Québec City QC Canada

**Keywords:** Fragile X mental retardation 1 protein, FXTAS, Latin America, Neurodegenerative diseases, Premutation

## Abstract

**Background:**

Fragile X–associated tremor/ataxia syndrome (FXTAS) is a late‐onset neurodegenerative disorder caused by *FMR1* premutation expansions (55–200 CGG repeats). Although well described in populations of predominantly European ancestry, FXTAS remains poorly characterized in Latin America due to limited awareness, restricted access to molecular diagnostics, and scarce epidemiological data.

**Objective:**

To synthesize available evidence on FXTAS and other *FMR1* premutation–associated phenotypes in Latin America, focusing on epidemiology, clinical and neuroimaging features, diagnostic approaches, and atypical presentations.

**Methods:**

A scoping review was conducted following PRISMA‐ScR guidelines. PubMed, EMBASE, Scopus, and LILACS were searched without date restrictions (last search: April 2025). Eligible studies reported FXTAS cases or *FMR1* premutation carriers from Latin American countries. Data were extracted on demographics, clinical manifestations, neuroimaging, molecular findings, and country of origin.

**Results:**

Twenty‐five studies including 5531 participants reported 38 FXTAS cases and 386 premutation carriers without FXTAS. Diagnoses of FXTAS occurred mainly between ages 53 and 85 years. Gait ataxia and tremor predominated, while parkinsonism, peripheral neuropathy, executive dysfunction, and psychiatric symptoms were also reported. Generalized cerebral atrophy was more frequent than the middle cerebellar peduncle sign. Atypical phenotypes, including spastic paraparesis and progressive supranuclear palsy–like presentations, contributed to diagnostic delays. Premutation carriers commonly exhibited fragile X–associated primary ovarian insufficiency and neuropsychiatric manifestations.

**Conclusions:**

FXTAS is likely underdiagnosed in Latin America despite recognizable clinical patterns and associated premutation‐related conditions. Improving clinician awareness, access to genetic testing, and standardized diagnostic evaluation is essential. Prospective regional studies are needed to define prevalence, penetrance, and gene–environment interactions.

## Introduction

Fragile X‐associated tremor/ataxia syndrome (FXTAS) is a progressive neurodegenerative disorder caused by premutation expansions (55–200 CGG repeats) in the *FMR1* gene.^1^ While the full mutation (>200 repeats) leads to Fragile X syndrome (FXS), a childhood‐onset neurodevelopmental disorder, the premutation gives rise to a heterogeneous spectrum of neurological and systemic conditions in adulthood, among which FXTAS is the most recognized.

In FXTAS, the *FMR1* premutation leads to toxic RNA accumulation and aberrant protein production via repeat‐associated non‐AUG (RAN) translation, resulting in neuronal damage, mitochondrial dysfunction, and the development of ubiquitin‐positive intranuclear inclusions within neurons and glial cells.^1^ Diagnosis relies on integrating clinical evaluation, genetic confirmation of the premutation, and neuroimaging.^1^, ^2^ Clinically, FXTAS is most often characterized by intention tremor, gait ataxia, parkinsonian features, and cognitive decline. Additional manifestations such as peripheral neuropathy, autonomic dysfunction, and psychiatric symptoms have also been reported, reflecting the variability of its presentation.^1^, ^2^ T2 hyperintensities in the middle cerebellar peduncles are considered the most distinctive radiological hallmark, although white matter changes in other regions and generalized brain atrophy are frequently observed.^3^

While the prevalence and impact of FXTAS have not been extensively studied in Latin America, several significant case reports and studies provide valuable insights into its occurrence and clinical features.^3^, ^4^ Colombia has one of the highest prevalences of FXS globally, particularly in the town of Ricaurte, Valle del Cauca, where the full mutation affects 1 in 19 men and 1 in 46 women, while the premutation is carried by 1 in 85 men and 1 in 25 women.^5^

Over the past two decades, the clinical spectrum associated with the *FMR1* premutation has expanded substantially, driven by increased awareness and wider access to molecular testing.^6^ Premutation alleles, once considered largely asymptomatic, are now known to be associated with several RNA toxicity–mediated conditions, including FXTAS,^7^ fragile X–associated primary ovarian insufficiency (FXPOI),^8^ and the more recently described fragile X–associated neuropsychiatric disorders (FXAND).^9^

Beyond these defined syndromes, *FMR1* premutation carriers may present with additional clinical manifestations that do not fulfill diagnostic criteria for FXTAS, FXPOI, or FXAND but occur at higher frequencies than in the general population. These include systemic and neurological features such as hypertension, myalgias, and peripheral neuropathy in the absence of classical FXTAS signs.^2^, ^10^ From a psychiatric perspective, premutation carriers also exhibit increased rates of depression and anxiety disorders, which have been linked to mitochondrial dysfunction, calcium dysregulation, oxidative stress, chronic DNA damage, and inclusion formation affecting limbic and frontal–subcortical circuits.^9^, ^11^, ^12^

Despite these advances, knowledge regarding FXTAS and other *FMR1* premutation–associated phenotypes in Latin America remains limited. Low clinical awareness, scarce epidemiological data, heterogeneous reporting, and restricted access to molecular diagnostics contribute to underrecognition and delayed diagnosis in the region. Accordingly, this scoping review aimed to synthesize the existing evidence on FXTAS in Latin America, providing an overview of reported epidemiological and clinical characteristics while highlighting current challenges and research gaps.

## Materials and Methods

We conducted a scoping review following the Preferred Reporting Items for Systematic Reviews and Meta‐Analyses Extension for Scoping Reviews (PRISMA‐ScR) guidelines.

### Eligibility Criteria

We included peer‐reviewed studies conducted in Latin American countries—defined as those in South America, Central America, and North America (with Mexico as the sole representative in the latter), as specified in the Supporting Information—that explicitly reported individuals carrying an *FMR1* premutation. Eligible studies described patients with FXTAS, other *FMR1* premutation–associated phenotypes, or premutation carriers without overt FXTAS manifestations.

FXTAS cases were included when diagnosis was reported according to specified clinical, molecular, and/or neuroimaging criteria. Observational studies (including cross‐sectional designs) and case reports were eligible for inclusion.

We excluded studies involving individuals with FXS associated with full *FMR1* CGG repeat expansions. Narrative reviews were excluded. Dissertations, conference proceedings, letters, and poster abstracts were also excluded. No restrictions were placed on publication date.

### Search Strategy

A systematic literature search was conducted in PubMed, EMBASE (Elsevier), Scopus, and LILACS. Database‐specific controlled vocabulary terms and free‐text keywords related to FXTAS, *FMR1* premutation, and Latin America were used. The full search strategies are detailed in Appendix S1. The final search was performed in April 2025.

After duplicate removal, two independent reviewers (AS and SPG) screened titles and abstracts for eligibility. Full texts of potentially relevant articles were subsequently assessed. Discrepancies were resolved by consensus and, when necessary, by consultation with a third reviewer (SM).

### Data Extraction and Analysis

Data extraction was independently performed by two reviewers (AS and SPG) using a standardized data extraction form. Extracted variables were defined a priori to correspond directly to the descriptive domains presented in Tables 1 and 2.

**TABLE 1:** Characteristics of individuals with an FXTAS diagnosis across Latin American studies

Study	Country	Study design	Number of patients with FXTAS/Total assessed (sex distribution)	Age of FXTAS diagnosis	*FMR1* mutation status of cohort/of FXTAS patients	# or range of CGG repeats in FXTAS subjects	Diagnostic methods	Clinical manifestations	Brain MRI findings
Almeida et al. (2024)^13^	Brazil	Case report	1/1 (1 male)	60 yo	1 PM/1 PM	100	PCR	1/1 ataxia, postural instability, spasticity	1/1 MCP sign, Hyperintensity in T2 (splenium of the corpus callosum
Ramírez ‐Cheyne et al (2024)^14^	Colombia	Cross sectional	4/ 55 (3 female 1 male)	Females: 53, 58, 60 yo Male: 85 yo	19 PM, 35 FM, 1 N/4 PM	55–200	PCR, Southern Blot	Not specified	Not specified
Dávila‐Ortiz de Montellano et al (2019)^15^	Mexico	Cross sectional	11/106 (5 males and 6 females)	Not specified	NR/7 PM, 3 GZ, 1 FM	PM: 124, 92, 56, 56, 87, 84 GZ: 48, 48, 52, 44 FM: >200	PCR	11/11 ataxia 4/11 intention tremor 4/11 parkinsonism 4/11 executive dysfunction 5/11 neuropathy	5/11 cerebral atrophy 1/11 MCP sign
Saldarriaga‐Gil et al (2019)^16^	Colombia	Cross sectional	4/25 (3 females 1 male)	Females: 54,80, 53 yo Male: 83 yo	25 PM/4 PM	55–200	PCR, Southern Blot	3/4 intention tremor 1/4 ataxia 2/4 seizures 2/4 anxiety	2/4 cerebral atrophy
Salomão et al (2019)^17^	Brazil	Case report	1/1 (1 female)	82 yo	1 PM/1 PM	75	PCR, Southern Blot	1/1 ataxia, parkinsonism, postural instability	1/1 Hummingbird sign
Saldarriaga‐Gil et al (2017)^18^	Colombia	Case report	1/1 (1 female)	53 yo	1 PM/1 PM	82	PCR, Southern Blot	1/1 tremor‐at‐rest, neuropathy, anxiety, cognitive impairment, FXPOI	1/1 cerebral atrophy
Dos Santos Ghilardi et al (2015)^19^	Brazil	Case report	1/1 (1 male)	65 yo	1 PM/1 PM	150–200	Clinical criteria, MRI, PCR and Southern blot	1/1 intention tremor, ataxia, dysarthria	1/1 cerebral atrophy
Santa María et al (2014)^4^	Chile	Case report	1/6 (1 male)	70 yo	2 PM, 4 FM/1 FM	180–410	PCR, Southern Blot and *FMR1* mRNA quantifications	1/1 intention tremor, ataxia, parkinsonism, postural instability, executive dysfunction, urinary incontinence, neuropathy, mood lability, hypersomnolence	1/1 cerebral atrophy
Jara ‐ Prado et al (2018)^20^	Mexico	Case series	1/3 (1 female)	65 yo	1 N, 1 PM, 1 FM/1 PM	87	PCR	1/1 Right‐sided intention tremor, resting tongue tremor, bradykinesia, oromandibular rigidity, mild mixed hearing loss, menopause at 41 years old.	1/1 normal
Alliende et al (2012)^21^	Chile	Case series	9/152 (sex not specified)	Not specified	58 PM, 63 FM, 6 Mo, 25 N/Not specified	Not specified	PCR, Southern Blot	Not specified	Not specified
Capelli et al (2007)^22^	Brazil	Case report	3/4 (3 males)	65, 73, 74 yo	3 PM, 1 N/3 PM	PM: 123, 109, 91 N: 30	PCR, Southern Blot	2/3 ataxia, dysmetria, dysdiadokokinesia, action tremor 1/3 intention tremor, parkinsonism, urinary incontinence	3/3 MCP sign, Cerebral atrophy
Gonçalves et al (2007)^23^	Brazil	Case report	1/1 (1 male)	70 yo	1 PM/1 PM	85	PCR	1/1 ataxia, postural instability, executive dysfunction, apathy, urinary incontinence	1/1 MCP sign, cerebral atrophy
Total			38/356 (14 males, 15 females, 9 not specified)		120 PM, 104 FM, 3 GZ, 28 N, 6 Mo, 95 NR (*n* = 356)/24 PM, 3 GZ, 2 FM, 9 Not specified (*n* = 38)				

Abbreviations: CGG, Cytosine‐guanine‐guanine trinucleotide repeat; FM, full mutation; *FMR1*, Fragile X mental retardation 1 gene; FXPOI, Fragile X‐associated primary ovarian insufficiency; FXTAS, Fragile X‐associated tremor/ataxia syndrome; GZ, gray zone; MCP, middle cerebellar peduncle; Mo, Mosaic; MRI, Magnetic resonance imaging; N, normal alleles; NR, Not reported; PCR, Polymerase chain reaction; PM, premutation; yo, years‐old.

**TABLE 2:** Characteristics of individuals carrying the *FMR1* premutation without FXTAS manifestations across Latin American studies

Study	Country	Study design	# of patients with *FMR1* premutation without FXTAS/Total assessed (sex distribution)	Diagnostic methods	Clinical manifestations other than FXTAS	Brain MRI findings
Ramírez‐Cheyne et al (2023)* ^14^	Colombia	Cross sectional	15/55 (3 males and 12 females) *4 with FXTAS	WES	3/19 with POI	NR
Saldarriaga et al (2021)^24^	Colombia	Cross sectional	118/1322 (22 males and 96 females)	Karyotype, PCR, Southern Blot, Exome	NR	NR
Cabal‐Herrera et al (2020)^25^	Colombia	Case report	1/1 (1 male)	NR	1/1 Alcohol abuse, irritability, delusional jealousy, depression and aggressive behavior	Mild frontal atrophy, mild ventriculomegaly and white matter disease in the periventricular regions
Ormazábal et al (2019)^26^	Argentina	Case series	22/55 (3 males and 19 females)	PCR, Southern Blot	3/19 females with POI, male asymptomatic.	NR
Ferreira et al (2019)^27^	Brazil	Cross sectional	4/90 (4 males)	PCR, Electrophoresis	4/4 non‐syndromic ASD	NR
Saldarriaga et al (2019)* ^16^	Colombia	Cross sectional	21/25 (5 males and 20 females) *4 with FXTAS	PCR, Southern Blot	1/25 with POI 1/25 with seizures 1/25 with cognitive deficit 2/25 with anxiety 1/25 with POI and seizures 1/25 with POI and anxiety 1/25 with cognitive deficit and anxiety 1/25 with seizures, cognitive deficit and anxiety 6/19 asymptomatic	NR
Miranda‐Furtado et al (2018)^28^	Brazil	Case control	4/121 (4 females)	PCR	4/4 with POI	NR
Santa María et al (2016)^29^	Chile	Retrospective cohort study	67/2321 (59 males, 8 females)	PCR, Southern Blot	6/67 (5 males and 1 females) with ID, rest not specified	NR
Santa María et al (2014)* ^4^	Chile	Case report	2/6 (2 females) *1 with FXTAS	PCR, Southern Blot	Not specified	NR
Alliende et al (2012)* ^21^	Chile	Case series	49/152 (39 females, rest not specified) *9 with FXTAS	PCR, Southern Blot	Not specified (17 females with POI and 9 subjects with FXTAS, genetic profile of these groups is not known)	NR
Yuhas et al (2009)^30^	Guatemala	Cross sectional	10/105 (2 males and 8 females)	PCR, Southern Blot	Not specified	NR
Reis et al (2008)^31^	Brazil	Cross sectional	1/66 (1 male)	PCR	1/1 Asymptomatic	NR
Barros‐Núñez et al (2008)^32^	Mexico	Cross‐sectional	8/540 (sex not specified)	PCR, Electrophoresis	Not specified	NR
Costa et al (2006)^33^	Brazil	Case control	23/157 (23 females)	PCR, Electrophoresis, Southern Blot	23/23 with POI	NR
Rosales‐Reynoso et al (2005)^34^	Mexico	Case control	3/129 (3 males)	PCR	3/3 Moderate ID	NR
Machado‐Ferreira et al (2002)^35^	Brazil	Cross sectional	6/26 (6 females)	Southern Blot	4/6 with POI	NR
Mingroni‐Netto et al (1996)^36^	Brazil	Cross sectional	32/242 (sex not specified)	PCR, Southern Blot	Not specified	NR
Total			386/5413 (103 males and 233 females, 50 not specified)			

* Patients with FXTAS were included but not counted in this table.

Abbreviations: ASD, Autism spectrum disorder; CGG, Cytosine‐guanine‐guanine trinucleotide repeat; *FMR1*, Fragile X mental retardation 1 gene; FXS, Fragile X syndrome; FXTAS, Fragile X‐associated tremor/ataxia syndrome; ID, Intellectual disability; MRI, Magnetic resonance imaging; NR, Not reported; PCR, Polymerase chain reaction; POI, Primary ovarian insufficiency; WES, Whole exome sequencing.

For each included study, we extracted first author and year of publication, country, and study design. For studies reporting FXTAS cases, additional extracted data included the number of individuals diagnosed with FXTAS and the total number of assessed participants, sex distribution, age at FXTAS diagnosis, *FMR1* mutation status in the assessed cohort, *FMR1* mutation status in patients with confirmed FXTAS, CGG repeat size, diagnostic methods used, reported clinical manifestations, and brain magnetic resonance imaging (MRI) findings.

For studies including *FMR1* premutation carriers without FXTAS manifestations, the same data fields were extracted when available, including number of carriers without FXTAS and total assessed participants, sex distribution, *FMR1* mutation status, diagnostic methods, reported clinical manifestations, and brain MRI findings. Variables not mentioned were recorded as not reported.

Discrepancies in data extraction were resolved through discussion and consensus. When required, a third reviewer (SM) was consulted to adjudicate disagreements. Due to heterogeneity in study designs, outcomes, and reporting practices, the results were synthesized descriptively, without quantitative meta‐analysis or formal risk of bias assessment.

In accordance with the PRISMA Extension for Scoping Reviews (PRISMA‐ScR), no formal risk of bias or methodological quality assessment was performed, as the primary objective of this review was to map the existing evidence, characterize reported phenotypes, and identify knowledge gaps rather than to evaluate intervention effects or generate pooled estimates.

## Results

### Study Selection

**Figure 1:**
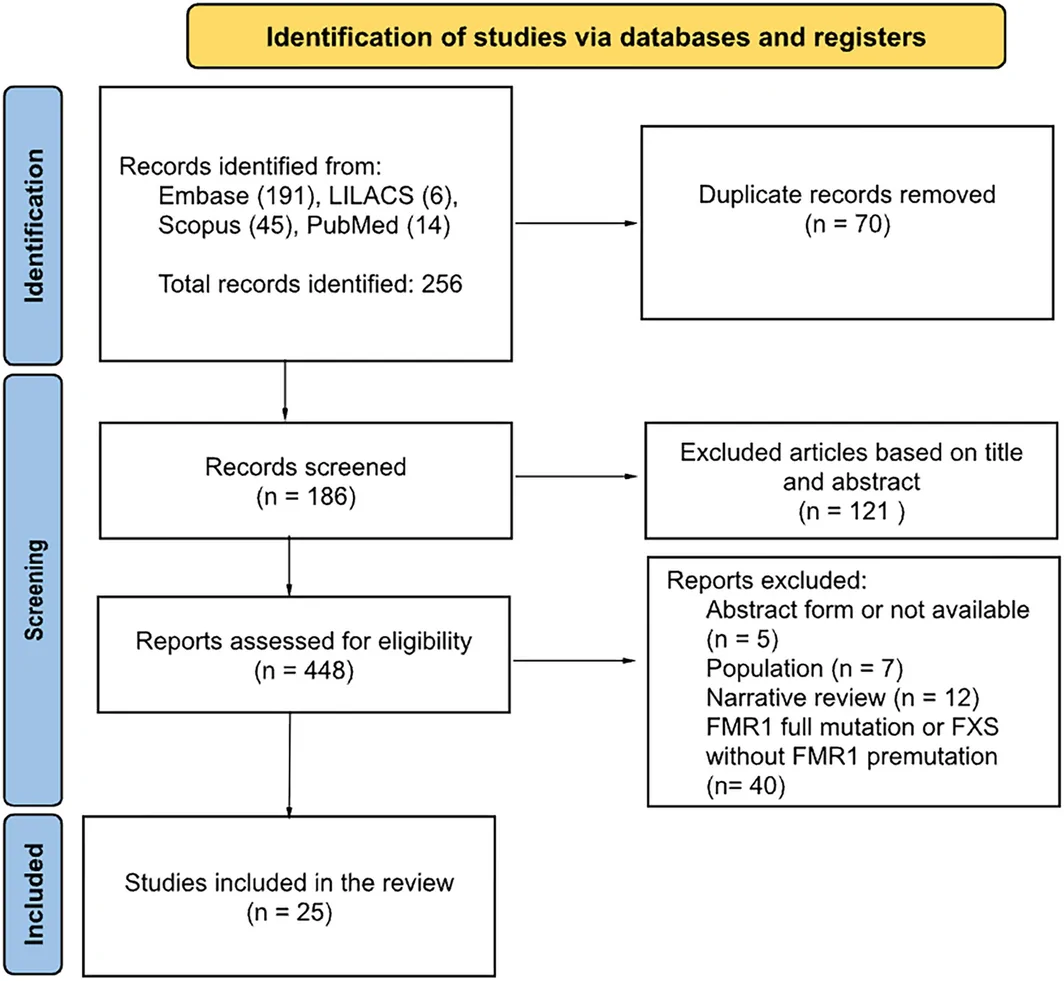
PRISMA flow diagram. A total of 256 records were identified from Embase, LILACS, Scopus, and PubMed. After removal of 70 duplicates, 186 titles and abstracts were screened, and 92 full texts were assessed for eligibility. After full text review, 25 studies from Latin America on *FMR1* premutation–associated phenotypes were included.

A total of 256 records were initially identified, of which 25 articles were assessed in full text. The main reasons for exclusion included duplicate entries, lack of full‐text availability (abstracts only), and failure to meet the inclusion criteria (non–Latin American populations, narrative reviews, or patients without FXTAS or *FMR1* premutation). The PRISMA flow diagram is shown in Figure 1.

### Overview of Included Studies and Populations

The 25 included studies comprised a total of 5531 screened participants. Among them, within the sample that had a *FMR1* expansion (premutation, gray‐zone or full‐mutation), 38 individuals had a confirmed diagnosis of FXTAS, and 386 were *FMR1* premutation carriers without clinical manifestations of FXTAS.

Twelve studies included patients with FXTAS,^4^, ^13^, ^14^, ^15^, ^16^, ^17^, ^18^, ^19^, ^20^, ^21^, ^22^, ^23^ seventeen studies enrolled *FMR1* premutation carriers without FXTAS,^4^, ^14^, ^16^, ^21^, ^24^, ^25^, ^26^, ^27^, ^28^, ^29^, ^30^, ^31^, ^32^, ^33^, ^34^, ^35^, ^36^ and four studies included both groups.^4^, ^14^, ^16^, ^21^ Regarding study design, three were case–control studies,^28^, ^33^, ^34^ three case series,^20^, ^21^, ^26^ eight case reports,^4^, ^13^, ^17^, ^18^, ^19^, ^22^, ^23^, ^25^ ten cross‐sectional studies,^14^, ^15^, ^16^, ^24^, ^27^, ^30^, ^31^, ^32^, ^35^, ^36^ and one retrospective cohort study.^29^

**Figure 2:**
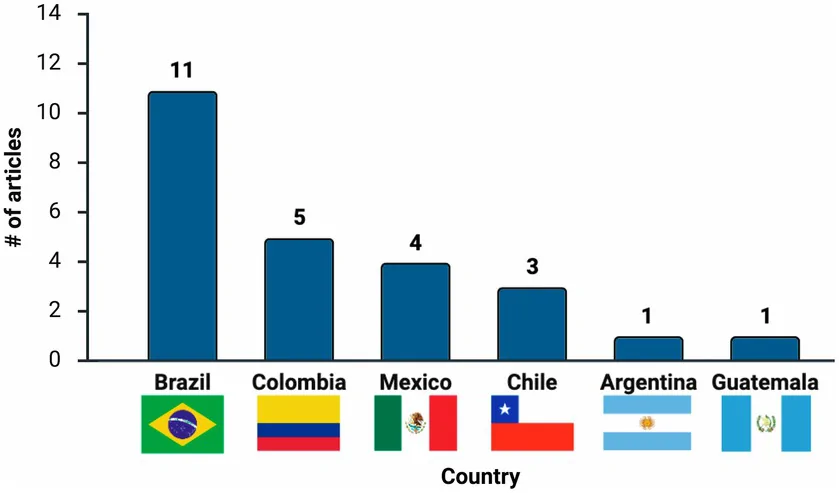
Geographic distribution of included studies by country. Bars represent the number of published studies per country. Brazil contributed the largest number of studies (*n* = 11), followed by Colombia (*n* = 5), Mexico (*n* = 4), and Chile (*n* = 3), with single studies from Argentina and Guatemala. Created in https://BioRender.com.

Geographically, studies originated from Colombia,^14^, ^16^, ^18^, ^24^, ^25^ Argentina,^26^ Brazil,^13^, ^17^, ^19^, ^22^, ^23^, ^27^, ^28^, ^31^, ^33^, ^35^, ^36^ Mexico,^15^, ^20^, ^32^, ^34^ Chile,^4^, ^21^, ^29^ and Guatemala.^30^ Brazil contributed the largest number of studies (*n* = 11), predominantly case reports and cross‐sectional studies, followed by Colombia (*n* = 5) and Mexico (*n* = 4). The geographic distribution of the included studies is schematically illustrated in Figure 2.

### FXTAS in Latin America

#### Epidemiology and Demographic Characteristics

Across the 12 studies that included FXTAS cases, 38 patients were identified. Specific age data were available for 18 of 38 patients (47.3% of the sample), with an age range of 53–85 years and a mean age of 66.83 years.

Both males and females were affected, although sex‐specific prevalence data were reported in only a limited number of studies (Table 1). One study conducted in Ricaurte, Colombia, highlighted that symptoms suggestive of FXTAS were observed in 50% of male carriers and 33.3% of female carriers aged over 50 years.^16^ Although potential environmental risk factors were infrequently assessed across studies, the Ricaurte study further reported that all affected carriers were engaged in agricultural work and had frequent exposure to pesticides through occupational activities, household storage, or contaminated food and water.^16^

#### *FMR1* Mutation Status and CGG Repeat Distribution

Genetic data showed substantial heterogeneity among FXTAS cases. Most patients were *FMR1* premutation carriers, with CGG repeat sizes ranging from 56 to 200 repeats.^13^, ^14^, ^15^, ^16^, ^17^, ^18^, ^19^, ^22^, ^23^ A small number of individuals carried gray zone alleles (44–52 CGG repeats),^15^ and full mutation alleles (>200 repeats).^4^, ^15^ Among the 120 patients with a confirmed *FMR1* premutation reported in the 12 studies that included FXTAS cases, 24 were reported to have FXTAS diagnosis (19.3%). Several studies did not report the exact CGG repeat number.^4^, ^14^, ^16^, ^19^, ^21^ A summary of genetic findings is presented in Table 1.

#### Clinical Manifestations Associated with FXTAS

Clinical manifestations were described in nine of the eleven studies, encompassing 24 FXTAS patients. The FXTAS phenotype was dominated by motor manifestations, particularly ataxia,^4^, ^13^, ^14^, ^15^, ^16^, ^17^, ^18^, ^19^, ^22^, ^23^ intention tremor^4^, ^15^, ^16^, ^20^, ^22^ and parkinsonian features.^4^, ^15^, ^17^, ^20^, ^22^ Additional motor findings included postural instability,^4^, ^13^, ^17^, ^23^ action tremor,^19^, ^22^ tremor at rest,^18^, ^20^ spasticity^13^ and cerebellar signs.^22^

Non‐motor manifestations were less frequently reported and included executive dysfunction,^4^, ^15^, ^23^ nonspecific cognitive impairment,^18^ autonomic symptoms such as urinary incontinence,^4^, ^22^, ^23^ mild hearing loss,^20^ neuropsychiatric features (including anxiety,^16^, ^18^ apathy,^23^ mood lability,^4^ and hypersomnolence^4^), and sensory symptoms such as peripheral neuropathy.^4^, ^15^, ^18^ Seizures and FXPOI were reported to be associated in isolated cases.^16^, ^18^ Detailed clinical features are summarized in Table 1.

#### Neuroimaging Findings

Neuroimaging data were available for 18 FXTAS patients across nine studies, with MRI being the most commonly used modality. The most frequently reported neuroimaging findings were cerebral atrophy (*n* = 10),^13^, ^15^, ^22^, ^23^ middle cerebellar peduncle (MCP) sign (*n* = 2),^13^, ^15^ and the coexistence of both findings (*n* = 4),^22^, ^23^ observed in approximately 33%, 11.1%, and 22.2% of patients with available MRI data, respectively. Splenial T2 hyperintensities were reported less often (*n* = 1),^13^ and one patient had normal MRI findings despite clinical criteria of FXTAS in the context of an *FMR1* premutation.^20^

Atypical imaging patterns were also reported, including cases presenting with alternative brainstem signs (hummingbird sign) in the absence of the classic MCP sign.^17^ Neuroimaging findings are summarized in Table 1.

#### Atypical FXTAS Presentations

Three case reports described atypical clinical presentations of FXTAS. One report detailed a male premutation carrier whose initial presentation was dominated by progressive spasticity and lower‐limb weakness, leading to an initial diagnostic consideration of hereditary spastic paraplegia. Neuroimaging later revealed bilateral MCP and splenial abnormalities, supporting a diagnosis of FXTAS with a spastic phenotype.^13^

Another case involved a male premutation carrier identified through familial screening who developed rapidly progressive cognitive decline with a frontal–subcortical profile, followed by gait ataxia and urinary incontinence, accompanied by typical FXTAS MRI findings.^23^

A third case described an elderly female premutation carrier with gait impairment and postural instability whose MRI showed a “hummingbird sign” and features suggestive of nigrostriatal involvement. Although initially interpreted as progressive supranuclear palsy, the overall phenotype was considered consistent with a PSP–pure akinesia with gait freezing phenotype in the context of *FMR1* premutation.^17^

#### Diagnostic Approaches

Diagnosis of FXTAS consistently relied on a combination of clinical evaluation, genetic testing, and neuroimaging. Several studies employed standardized clinical scales, including the Scale for the Assessment and Rating of Ataxia (SARA) and the Fahn–Tolosa–Marín tremor scale,^16^, ^18^, ^19^ the International Cooperative Ataxia Rating Scale (ICARS),^16^, ^22^ and the Unified Parkinson's Disease Rating Scale (UPDRS).^22^

Genetic testing of the *FMR1* gene was performed in all studies, most commonly using polymerase chain reaction (PCR) and Southern blot techniques to determine CGG repeat length and, in some cases, methylation status.^4^, ^13^, ^14^, ^15^, ^16^, ^17^, ^18^, ^19^, ^20^, ^21^, ^22^, ^23^ Less frequently, additional complementary approaches, such as *FMR1* mRNA quantification, were used to assess gene expression but not as primary diagnostic methods.^4^ One study evaluating revised FXTAS diagnostic criteria showed a sensitivity of 36% and a specificity of 43%, with a positive predictive value of 7% and a negative predictive value of 85%.^15^

#### Treatment and Management

Treatment approaches were predominantly symptomatic. Pharmacological data were reported in only two studies. In one case, levodopa was administered at a dose of 800 mg/day without clinical improvement. In another report, the patient was treated with propranolol, primidone, and clonazepam, also without significant symptomatic benefit. No specific pharmacological treatment details were reported in the remaining studies.

One study reported the use of deep brain stimulation targeting the ventral oral posterior nucleus and zona incerta in a patient with severe tremor and ataxia, resulting in sustained motor improvement over 30 months without adverse effects.^4^

Additionally, the importance of specialized multidisciplinary centers was emphasized, integrating genetic diagnosis and counseling with rehabilitative therapies and facilitating early recognition of FXTAS among premutation carriers.^15^

#### Summary of Findings in Latin American Cohorts Including Patients with FXTAS

Table 3 provides a structured summary of the key epidemiological, genetic, clinical, and neuroimaging findings identified among Latin American patients diagnosed with FXTAS, synthesized from the data presented in Tables 1 and 2. The table highlights both the principal patterns and atypical features observed across cohorts, offering a consolidated reference for the most relevant and unusual findings.

**TABLE 3:** Key epidemiological, clinical, and radiological findings in Latin American FXTAS cohorts

Variable	Patients with FXTAS diagnosis
Demographics	Screened participants: 5531 FXTAS cases (n = 38 confirmed) Sex (data available for 29 patients) Male: 14/29 (48.3%) Female: 15/29 (51.7%) Age (data available for 18 patients) Mean (range): 66.8 (53–85)
Environmental exposure	Occupational pesticide exposure: 4/38 (10.5%) From a single Colombian cluster
Genetic status	*FMR1* status (data available for 29 patients) PM: 24/29 (82.7%) GZ: 3/29 (10.4%) FM: 2/29 (6.9%)
Clinical phenotype*	Ataxia: 19/38 (50%) Intention tremor: 11/38 (29%) Parkinsonism features: 7/38 (18.4%) Postural instability: 4/38 (10.5%) Neuropathy: 7/38 (18.4%) Executive dysfunction / Cognitive impairment: 7/38 (18.4%) Other neuropsychiatric symptoms (eg, apathy, anxiety, mood lability): 5/38 (13.2%)
Atypical presentations	Spastic paraparesis: 1/38 (2.6%) Rapidly progressive dementia: 1/38 (2.6%) PSP‐like phenotype: 1/38 (2.6%)
Neuroimaging findings	Brain MRI (data available for 18 patients) Cerebral atrophy: 10/18 (55.6%) MCP sign: 2/18 (11.1%) Cerebral atrophy + MCP sign: 4/18 (22.2%) Normal/nonspecific: 2/18 (11.1%)

* Clinical variables were non–mutually exclusive.

Abbreviations: FM, Full mutation; GZ, Gray zone; MCP, Middle cerebellar peduncle; MRI, Magnetic resonance imaging; PM, Premutation; PSP, Progressive supranuclear palsy.

### *FMR1* Premutation Carriers without FXTAS in Latin America

#### Population Characteristics

Across the 17 studies that included *FMR1* premutation carriers without FXTAS, 386 individuals were identified among 5413 assessed participants. Sex was reported for most individuals, with a predominance of females (233 females, 103 males), while sex was not specified in 50 cases. Four of the 17 studies reporting *FMR1* premutation carriers without FXTAS provided specific age data, corresponding to 41 of the 386 patients (10.6%). Among these 41 patients, the age range was 18–80 years, with a mean age of 45.29 years. Many carriers were identified through family screening of individuals with FXS. A complete description of demographic characteristics for each study is provided in Table 2.

#### Diagnostic Approaches

Genetic diagnosis was performed primarily using PCR and Southern blot analysis, either alone or in combination.^4^, ^16^, ^20^, ^21^, ^24^, ^26^, ^27^, ^28^, ^29^, ^30^, ^31^, ^32^, ^33^, ^34^, ^35^, ^36^ Capillary electrophoresis was used in several studies to refine CGG repeat sizing.^27^, ^32^, ^33^ Whole Exome Sequencing (WES) and karyotype analysis were reported in a minority of studies.^14^, ^24^ In some studies, the diagnostic method was not specified.

#### Clinical Manifestations outside the FXTAS Spectrum

Clinical features distinct from FXTAS were described in 13 studies.^14^, ^16^, ^20^, ^25^, ^26^, ^27^, ^28^, ^29^, ^32^, ^33^, ^34^, ^35^, ^36^ The most frequently reported condition was FXPOI, affecting 40 women.^14^, ^16^, ^21^, ^26^, ^28^, ^33^, ^35^ Other reported manifestations included intellectual disability,^16^, ^29^, ^34^ autism spectrum disorder,^27^ and neuropsychiatric symptoms such as anxiety, irritability, and depression.^16^, ^25^ Most carriers were asymptomatic or lacked detailed clinical descriptions.

#### Neuroimaging Findings

Neuroimaging was reported in only one premutation carrier without FXTAS, showing mild frontal atrophy, ventriculomegaly, and periventricular white matter changes in association with neuropsychiatric symptoms.^25^ No additional imaging data were available. A summary of findings is provided in Table 2.

## Discussion

This scoping review provides the most comprehensive synthesis to date of FXTAS and *FMR1* premutation–associated phenotypes reported in Latin America. Overall, the clinical and radiological features of FXTAS described in the region are broadly consistent with those reported in cohorts of predominantly European ancestry. However, several region‐specific patterns emerge that are relevant for clinical recognition and epidemiological interpretation.

Across 25 studies comprising 5531 screened participants, only 38 individuals were diagnosed with FXTAS despite the presence of large cohorts of reported *FMR1* premutation carriers (*n* = 506). This apparent underrecognition likely reflects the insidious and slowly progressive nature of *FMR1* mRNA–mediated neurotoxicity, which delays overt clinical expression, together with limited access to molecular testing, low clinical awareness of FXTAS, and substantial phenotypic heterogeneity.^6^, ^37^, ^38^ These factors are particularly relevant when early, atypical, or non‐classical manifestations predominate, increasing the likelihood that FXTAS remains unrecognized or misclassified.

A clear age contrast emerged between individuals with FXTAS and *FMR1* premutation carriers without overt neurological involvement. Among patients with FXTAS for whom age data were available, the mean age was 66.83 years (range 53–85), whereas *FMR1* premutation carriers without FXTAS had a substantially lower mean age of 45.29 years (range 18–80). Although age information was not available for the entire sample, this difference is consistent with the established age‐related increase in penetrance of FXTAS. In male premutation carriers, penetrance increases markedly with age, ranging from approximately 17% in those aged 50–59 years to up to 75% in individuals aged ≥80 years^39^ (approximately 40–45% across age groups).^40^ Data in females are more limited; however, penetrance of FXTAS appears consistently lower than in males, estimated at approximately 16% in comparable age ranges.^40^ These findings support the interpretation that the lower frequency of FXTAS observed in this regional sample may partly reflect the age structure of the studied cohorts, particularly among carriers identified through family screening at younger ages.

A notable observation was the relatively balanced sex distribution among reported FXTAS cases. This contrasts with data from cohorts of predominantly European ancestry, where the *FMR1* premutation is more prevalent in women (approximately 1 in 129–151 females vs. 1 in 468–649 males),^41^, ^42^ yet FXTAS shows a clear male predominance.^6^, ^38^ This pattern has been attributed to the higher age‐dependent penetrance observed in males, particularly after 50 years of age.^39^, ^43^, ^44^ This finding should be interpreted cautiously given small sample sizes, incomplete sex reporting, and the predominance of case‐based designs. Additionally, regional factors, including founder‐effect populations with high *FMR1* expansion frequencies in both sexes,^5^ may influence ascertainment patterns without implying equivalent biological penetrance.

Environmental exposures, including chronic contact with neurotoxic agricultural pesticides reported in some communities, have been proposed as potential modifiers of disease expression; however, supporting evidence is still scarce.^16^ Reports outside Latin America have described earlier‐than‐expected FXTAS onset in premutation carriers chronically exposed to organochlorine pesticides, industrial solvents such as toluene diisocyanate and dichloromethane, hexachlorocyclopentadiene‐related compounds, and herbicides.^45^ Additionally, chemotherapy exposure (eg, carboplatin) has also been associated with transient worsening of tremor and ataxia in susceptible carriers.^46^ Collectively, these observations underscore the importance of further investigation into environmental factors that may modulate FXTAS penetrance in Latin America.

From a molecular perspective, most FXTAS cases involved premutation alleles, but individuals with gray‐zone and full‐mutation alleles were also identified. This observation aligns with emerging evidence from other regions indicating that FXTAS‐like phenotypes can occur outside the classical premutation range,^14^, ^47^ particularly in mosaic or partially methylated contexts.^1^

Gray‐zone *FMR1* alleles have been associated with parkinsonism,^48^ a phenotype traditionally considered a minor feature within the FXTAS spectrum, and gray‐zone expansions have even been reported to be associated with Parkinson's disease (PD).^49^ In this cohort, one of three gray‐zone carriers (female) presented parkinsonism, consistent with prior reports suggesting increased susceptibility in gray‐zone carriers, particularly women.^48^ Findings across cohorts support a clinical continuum across *FMR1* CGG expansion ranges. Although the small number of gray‐zone cases precludes genotype–phenotype inference, this variability highlights the limitations of strict molecular cutoffs and supports a clinically driven diagnostic approach, consistent with expanded criteria recognizing that any pathogenic *FMR1* expansion may confer FXTAS risk when clinical suspicion is high.^50^

Clinically, FXTAS presentations in Latin America closely resemble those reported elsewhere, with gait ataxia and intention tremor as the most frequent motor features.^50^, ^51^ Parkinsonian signs were also commonly described, underscoring the potential for clinical overlap with idiopathic PD or atypical parkinsonian syndromes.^52^ This overlap is clinically relevant, as it may contribute to misdiagnosis in older adults when *FMR1* testing is not routinely considered. Non‐motor manifestations, including peripheral neuropathy,^15^, ^53^ executive dysfunction,^4^, ^15^, ^18^, ^23^ and neuropsychiatric symptoms,^16^, ^18^, ^23^ were frequently reported and further emphasize the multisystem nature of FXTAS. Importantly, accumulating evidence indicates that the phenotypic expression of the *FMR1* premutation differs between men and women across the entire clinical spectrum, extending beyond penetrance rates to differences in neurological, cognitive, psychiatric, and radiological manifestations. However, sex‐stratified analyses were not feasible in our sample due to inconsistent and incomplete reporting of clinical manifestations by sex across the included studies.

Several cases exhibited atypical presentations, including spastic paraparesis,^13^ rapidly progressive cognitive decline,^23^ and phenotypes resembling progressive supranuclear palsy.^17^ These reports illustrate that FXTAS may initially involve non‐cerebellar neural systems,^38^ potentially delaying recognition when classical tremor–ataxia features are absent. Atypical neuroimaging findings, such as non‐MCP white matter changes or isolated cerebellar atrophy, further complicate diagnosis.^6^ Reports from cohorts outside Latin America have additionally described rare movement disorders such as chorea or generalized reflex myoclonus as a manifestation of FXTAS, although they are not considered core features.^54^, ^55^, ^56^ Retrospective analyses highlight substantial diagnostic heterogeneity prior to FXTAS recognition, with common initial diagnoses including parkinsonism (24%), tremor (20%), ataxia (17%), dementia (13%), and cerebrovascular disease (10%).^57^

Neuroimaging findings across studies were heterogeneous. The MCP sign was reported in only a minority of cases,^13^, ^15^, ^22^, ^23^ whereas generalized cerebral atrophy was more frequently described.^4^, ^15^, ^16^, ^18^, ^19^, ^22^, ^23^ Normal or nonspecific MRI findings were also reported in clinically compatible cases, consistent with previous observations that imaging abnormalities may be absent in early disease stages or in women.^58^ These findings reinforce that neuroimaging should be interpreted as a supportive, rather than definitive, diagnostic component.^1^

Diagnostic approaches were broadly aligned with international recommendations, combining clinical assessment, molecular testing, and neuroimaging.^58^ However, standardized rating scales and formal diagnostic criteria were inconsistently applied, particularly in atypical cases. In some reports, a diagnosis of FXTAS was assigned despite incomplete fulfillment of classical radiological criteria (eg, absence of the MCP sign), and in a few instances the diagnosis was extended to individuals with gray‐zone alleles or unmethylated full‐mutation expansions rather than strictly within the premutation range. These decisions likely reflected a clinically driven approach in the context of suggestive neurological features and documented *FMR1* expression. Some studies incorporated standardized rating scales such as SARA, ICARS, FTM, and MDS‐UPDRS (part III) to quantify motor and ataxic symptoms.^16^, ^18^, ^19^, ^22^ This variability, together with limited diagnostic resources,^15^ likely contributes to underrecognition and incomplete phenotypic characterization in the region.

Beyond FXTAS, we identified 386 *FMR1* premutation carriers without an FXTAS phenotype, many of whom presented other premutation–associated conditions, including FXPOI, intellectual disability, autism spectrum disorder, and affective or behavioral disturbances such as anxiety, irritability, and depression. The predominance of FXPOI among female carriers—affecting approximately 20% before age 40, particularly in those with 75–100 CGG repeats^51^—along with recurrent neuropsychiatric manifestations, aligns with the broader framework of *FMR1* premutation–associated disorders (FXPOI, FXTAS, FXAND), thought to arise from shared mechanisms including RNA toxicity, mitochondrial dysfunction, and chronic cellular stress.^11^ However, clinical characterization in Latin American reports was often incomplete, with limited sex and age data, no neuroimaging report, and heterogeneous molecular diagnostic methods, particularly among asymptomatic or mildly symptomatic individuals identified through family screening. These limitations likely contribute to underrecognition of the true clinical burden and underscore a critical gap in systematically defining the penetrance and full spectrum of premutation‐associated manifestations in the region. In this context, the underrepresentation of Latin American populations in human genetics research may further limit the generalizability of genotype–phenotype associations derived largely from predominantly European‐ancestry cohorts. Our findings underscore the need for systematic, regionally grounded studies of FXTAS and other *FMR1* premutation–associated phenotypes.

### Strengths and Limitations

This scoping review provides the first regionally focused synthesis of FXTAS and *FMR1* premutation–associated conditions in Latin America, integrating evidence across countries with important genetic and environmental particularities. A comprehensive search strategy across multiple databases strengthened the inclusion of studies from diverse settings, and the structured extraction of clinical, genetic, and neuroimaging data enabled a multidimensional characterization of affected individuals.

However, several limitations must be acknowledged. Most available studies were case reports or small observational cohorts, limiting the precision of prevalence estimates and reducing the strength of causal inferences. Clinical documentation was heterogeneous and typically incomplete, particularly regarding neuropsychological and autonomic assessments, which may underestimate the true extent of non‐motor involvement. Neuroimaging findings were inconsistently reported and frequently limited to qualitative descriptions. Age was not systematically reported for all patients, restricting more robust comparisons between FXTAS and non‐FXTAS premutation carriers and limiting inferences regarding age‐dependent penetrance. Additionally, selection bias is likely, since individuals undergoing genetic testing in Latin America often originate from clinically complex or familial cases rather than representative population samples. Finally, the absence of standardized diagnostic criteria for premutation–associated neuropsychiatric and systemic manifestations restricts the ability to delineate the full clinical spectrum in asymptomatic or mildly symptomatic carriers.

### Future Directions

Addressing these gaps will require coordinated regional research efforts. Prospective, longitudinal cohort studies with standardized neurological, cognitive, psychiatric, and imaging evaluations are essential to clarify disease trajectories and penetrance, particularly among female carriers and individuals with gray‐zone or full‐mutation expansions. Environmental and occupational exposure assessments should be incorporated to better characterize potential gene–environment interactions, especially in agricultural communities with known neurotoxic risks.

Expanding access to molecular diagnostics and specialized neurological care is also crucial to reduce diagnostic delays and capture earlier disease stages. Screening programs targeting high‐risk families and founder‐effect populations could improve case detection and enhance genetic counseling infrastructure. Harmonization of diagnostic criteria and systematic surveillance across Latin America would facilitate inter‐country comparisons and guide public health strategies. Future reports should also ensure systematic documentation of key clinical and molecular variables, including age, sex, CGG repeat number (range and mean), and pharmacological treatments used in patients with FXTAS. Standardized reporting of these variables would strengthen genotype–phenotype correlations, allow more accurate age‐stratified penetrance estimates, and improve comparability across cohorts. Finally, translational research exploring biomarkers, modifiable risk factors, and emerging therapeutic interventions may contribute to more precise and preventive approaches to *FMR1*–associated neurodegeneration in the region.

## Conclusions

This scoping review demonstrates that FXTAS is reported in Latin America with clinical, radiological, and molecular features largely consistent with global descriptions, but with notable regional particularities, including a more balanced sex distribution and frequent atypical presentations. FXTAS appears to be underrecognized in the region, and phenotypic heterogeneity likely contributes to diagnostic delay.

Given the broad spectrum of *FMR1* premutation–associated manifestations, FXTAS should be considered in the differential diagnosis of late‐onset movement, parkinsonian, or cognitive disorders. Diagnosis should rely on integrated clinical assessment, molecular testing, and neuroimaging rather than on classical phenotypes alone. Improving access to molecular diagnostics and increasing clinician awareness are key steps to reduce missed or delayed diagnoses. Future studies should prioritize prospective, population‐based approaches to better define prevalence, penetrance, and modifiers of disease expression in Latin America.

## Author Roles

(1) Research project: A. Conception, B. Organization, C. Execution; (2) Statistical Analysis: A. Design, B. Execution, C. Review and Critique; (3) Manuscript Preparation: A. Writing of the first draft, B. Review and Critique;

A.S.: 1B, 1C, 2B, 2C, 3A, 3B

S.P.G.: 1B, 1C, 2B, 2C, 3A, 3B

S.M.: 1C, 2B, 2C, 3A

J.R.T.: 1C, 2B, 2C, 3A

K.O.V.: 1C, 2A, 3B

A.R.F.: 2C, 3B

C.C.C.: 1A, 1B, 2C, 3B

## Disclosures

**Ethical Compliance Statement:** This scoping review does not involve human participants or identifiable data and, therefore, does not require approval from an ethics committee or informed consent. We confirm that we have read the Journal's position on issues involved in ethical publication and affirm that this work is consistent with those guidelines.

**Funding Sources and Conflicts of Interest:** The authors received no financial support for the research, authorship, or publication of this article. All authors meet the authorship criteria as defined by the International Committee of Medical Journal Editors (ICMJE), declare that they have no conflicts of interest, and confirm that no related papers from the same study, nor any similar manuscripts, have been published elsewhere.

## Financial Disclosures and Conflicts of Interest

Author disclosures are available in the Supporting Information.

## Supporting information


**Appendix S1.** Search strategy. Database‐specific search strategies for FXTAS/*FMR1* in humans across Latin America, detailing controlled vocabulary and keywords used in Embase, PubMed, LILACS, and Scopus.

**Data S1.** COI_disclosure.

## Data Availability

The data analyzed in this scoping review were derived from publicly available sources cited in the article.
