# Supplementary material for: FXTAS and the Spectrum of FMR1 Premutation‐Associated Phenotypes in Latin America: A Scoping Review
Source: Mov Disord Clin Pract. 2026 Apr 5;13(9):2045–58. doi: 10.1002/mdc3.70617 (PMC13339662; doi:10.1002/mdc3.70617)
Supplement: Supplementary file 2 [file MDC3-13-2045-s002.docx]

**Appendix A. Search strategy**

**Embase**

('fragile x tremor ataxia syndrome'/exp OR FXTAS:ti,ab,kw OR FMR1:ti,ab,kw) AND [humans]/lim AND ('argentina' OR 'belize' OR 'bolivia' OR 'brazil' OR 'chile' OR 'colombia'

OR 'costa rica' OR 'cuba' OR 'dominica' OR 'dominican republic' OR 'ecuador' OR 'el salvador' OR 'grenada' OR 'guatemala' OR 'guyana' OR 'honduras' OR 'haiti' OR 'jamaica' OR 'mexico' OR 'nicaragua' OR 'panama' OR 'paraguay' OR 'peru' OR 'saint lucia' OR 'suriname' OR 'saint vincent and the grenadines' OR 'uruguay' OR 'venezuela' OR 'latin america')

**PubMed**

(("Fragile X Tremor Ataxia Syndrome"[MeSH Terms] OR FXTAS OR FMR1) AND Humans AND (Argentina OR Belize OR Bolivia OR Brazil OR Chile OR Colombia OR Costa Rica OR Cuba OR Dominica OR Dominican Republic OR Ecuador OR El Salvador OR Grenada OR Guatemala OR Guyana OR Honduras OR Haiti OR Jamaica OR Mexico OR Nicaragua OR Panama OR Paraguay OR Peru OR Saint Lucia OR Suriname OR Saint Vincent and the Grenadines OR Uruguay OR Venezuela OR "Latin America"))

**LILACS**

("Fragile X Tremor Ataxia Syndrome" OR FXTAS OR "FMR1") AND Humans AND (Argentina OR Belize OR Bolivia OR Brazil OR Chile OR Colombia OR "Costa Rica" OR Cuba OR Dominica OR "Dominican Republic" OR Ecuador OR "El Salvador" OR Grenada OR Guatemala OR Guyana OR Honduras OR Haiti OR Jamaica OR Mexico OR Nicaragua OR Panama OR Paraguay OR Peru OR "Saint Lucia" OR Suriname OR "Saint Vincent and the Grenadines" OR Uruguay OR Venezuela OR "Latin America")

**Scopus**

TITLE-ABS-KEY("Fragile X-associated tremor/ataxia syndrome" OR "Fragile X associated tremor ataxia syndrome" OR "Fragile X tremor ataxia syndrome" OR FXTAS OR FMR1)

AND

TITLE-ABS-KEY(Argentina OR Belize OR Bolivia OR Brazil OR Chile OR Colombia

OR "Costa Rica" OR Cuba OR Dominica OR "Dominican Republic" OR Ecuador OR "El Salvador" OR Grenada OR Guatemala OR Guyana OR Honduras OR Haiti OR Jamaica OR Mexico OR Nicaragua OR Panama OR Paraguay OR Peru OR "Saint Lucia" OR Suriname OR "Saint Vincent and the Grenadines" OR Uruguay OR Venezuela OR "Latin America")
